# Coping strategies towards mobbing used by medical and nursing staff in the hospitals of the prefecture of Etoloakarnania, Greece

**DOI:** 10.1192/j.eurpsy.2022.2291

**Published:** 2022-09-01

**Authors:** M. Theodoratou, A. Fasoulas, I. Farmakopoulou, K. Flora, G. Tsitsas, G. Kougioumtzis

**Affiliations:** 1 Hellenic Open University, Humanistic Sciences, Patras, Greece; 2 Neapolis University of Pafos, Health Sciences, Pafos, Cyprus; 3 Hellenic Open University, Human Sciences, PAtras, Greece; 4 University of Patras, Humanistic Sciences, Patras, Greece

**Keywords:** mobbing, Healthcare professionals, coping strategies

## Abstract

**Introduction:**

The aim of this study is to investigate mobbing in the Hospitals of Etoloakarnania, Greece. Additionally, this research aims to find out which coping strategies are used by health care professionals who have suffered mobbing and how their quality of life has been affected.

**Objectives:**

This research aims to depict mobbing phenomenon’s extent and investigate the coping strategies and quality of life of working staff, victims of mobbing.

**Methods:**

The research methodology was based on two questionnaires: LIPT scale and the scale of assessing bullying management strategies, as well as demographic data, which were answered by 130 people. A sufficient sample for the needs of this study to produce comparable results with the existing literature.

**Results:**

Mobbing is observed to a large extent of 83.8% , which, however, seems to be at lower levels in relation to corresponding researches abroad. In addition, the majority of respondents who appear to have been harassed at work are mostly women (78.5%), which seems to be in line with global studies. Τhe consultants and the supervisors take advantage of their position of power and impose or change duties on other staff (45.3%), in order to punish their subordinates, exacerbating the phenomenon of mobbing, also. Finally, the participants recognize the phenomenon and look for the majority of positive ways of action (65%), while they do not resort to non-constructive ways of solving the problem.

**Conclusions:**

Mobbing is a serious phenomenon that affects working staff’s mental health and quality of life. Policies should address effectively this deleterious aggressive behaviour.

**Disclosure:**

No significant relationships.

